# Short-term strength and balance training does not improve quality of life but improves functional status in individuals with diabetic peripheral neuropathy: a randomised controlled trial

**DOI:** 10.1007/s00125-019-04979-7

**Published:** 2019-08-29

**Authors:** Kavita Venkataraman, Bee Choo Tai, Eric Y. H. Khoo, Subramaniam Tavintharan, Kurumbian Chandran, Siew Wai Hwang, Melissa S. L. A. Phua, Hwee Lin Wee, Gerald C. H. Koh, E. Shyong Tai

**Affiliations:** 1 Saw Swee Hock School of Public Health, National University of Singapore and National University Health System, Tahir Foundation Building (MD1), 12 Science Drive 2, Singapore, 117549 Republic of Singapore; 2 Department of Medicine, Yong Loo Lin School of Medicine, National University of Singapore and National University Health System, Singapore, Republic of Singapore; 3 Diabetes Centre, Khoo Teck Puat Hospital, Singapore, Republic of Singapore; 4 Department of Medicine, Ng Teng Fong General Hospital, Singapore, Republic of Singapore; 5 SingHealth Polyclinics-Bukit Merah, Singapore, Republic of Singapore; 6 Allied Health Services and Pharmacy, Foot Care and Limb Design Centre, Podiatry Service, Tan Tock Seng Hospital, Singapore, Republic of Singapore; 7 Department of Pharmacy, Faculty of Science, National University of Singapore, Singapore, Republic of Singapore

**Keywords:** Balance, Diabetes, Diabetic neuropathy, Functional ability, Muscle strength, Physical therapy, Quality of life

## Abstract

**Aims/hypothesis:**

The aim of this study was to test the effectiveness of a structured strength and balance training intervention in improving health-related quality of life (HRQoL) and functional status in individuals with diabetic peripheral neuropathy (DPN).

**Methods:**

The study was a single-blind parallel-group randomised controlled trial comparing 2 months of once-weekly home-based strength and balance training against standard medical therapy. Participants were patients with physician-diagnosed type 2 diabetes and neuropathy recruited from five public sector institutions in Singapore between July 2014 and October 2017. Participants were block-randomised to intervention or control arms. Outcomes were assessed at baseline, 2 months and 6 months by a trained assessor blinded to group assignment. Primary outcomes were change in physical component summary (PCS) score of SF-36v2 (a 36-item generic HRQoL instrument that has been validated for use in Singapore) and EQ-5D-5L index score (derived from a five-item generic HRQoL instrument [EQ-5D-5L]) over 6 months. Secondary outcomes were change in functional status (timed up-and-go [TUG], five times sit-to-stand [FTSTS], functional reach, static balance, ankle muscle strength and knee range of motion) and balance confidence over 6 months. Mean differences in scores between groups were compared using mixed models.

**Results:**

Of the 143 participants randomised (intervention, *n* = 70; control, *n* = 73), 67 participants were included in each arm for the final intention-to-treat analysis. The two groups were similar, except in terms of sex. There were no significant differences between groups on the primary outcomes of PCS score (mean difference [MD] 1.56 [95% CI −1.75, 4.87]; *p* = 0.355) and EQ-5D-5L index score (MD 0.02 [95% CI −0.01, 0.06]; *p* = 0.175). There were significant improvements in TUG test performance (MD −1.14 [95% CI −2.18, −0.1] s; *p* = 0.032), FTSTS test performance (MD −1.31 [95% CI −2.12, −0.51] s; *p* = 0.001), ankle muscle strength (MD 4.18 [95% CI 0.4, 7.92] N; *p* = 0.031), knee range of motion (MD 6.82 [95% CI 2.87, 10.78]°; *p* = 0.001) and balance confidence score (MD 6.17 [95% CI 1.89, 10.44]; *p* = 0.005). No adverse events due to study participation or study intervention were reported.

**Conclusions/interpretation:**

Short-term structured strength and balance training did not influence HRQoL but produced sustained improvements in functional status and balance confidence at 6 months. More intensive interventions may be needed to influence HRQoL in these individuals. However, this intervention may be a useful treatment option for individuals with DPN to reduce the risk of falls and injuries.

**Trial registration:**

ClinicalTrials.gov NCT02115932

**Funding:**

This work was supported by the National Medical Research Council, Singapore.

**Electronic supplementary material:**

The online version of this article (10.1007/s00125-019-04979-7) contains peer-reviewed but unedited supplementary material, which is available to authorised users.

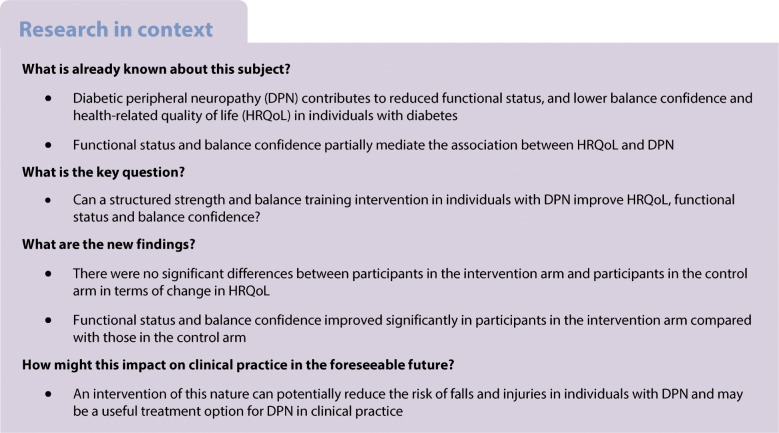



## Introduction

The most common neuropathy associated with diabetes mellitus is distal symmetrical polyneuropathy, with a glove-and-stocking distribution of sensory and motor loss [1]. This form of neuropathy, also known as diabetic peripheral neuropathy (DPN), affects around 12–50% of individuals with diabetes mellitus at any given point in time [2, 3]. DPN is a known important risk factor for serious adverse sequelae such as foot ulceration and amputation [4, 5]. Equally importantly, DPN itself is symptomatically distressing to individuals [6] and is associated with a reduction in health-related quality of life (HRQoL).

Classically, associations between DPN and HRQoL have been studied with respect to positive symptoms, especially pain [7–9]. Most of the randomised controlled trials for improvement of HRQoL in individuals with DPN have also focused on relief from neuropathic pain. However, there is increasing recognition that DPN is associated with reduced HRQoL, even without pain [2, 10]. We and others have demonstrated that DPN affects all domains of HRQoL, specifically physical functioning and physical role domains of the SF-36 [2, 10, 11]. In previous work, we found that DPN had the strongest association with reduced scores in the physical domains of HRQoL in individuals with diabetes-related complications, when compared with diabetic individuals without any complications [2].

It is well known that DPN contributes to reduced functional performance in individuals with diabetes [12, 13]. Those with DPN have been found to have reduced proprioceptive sense [14], reduced ankle mobility and range of motion [15, 16] and decreased muscle strength [15], especially in ankle and foot plantar and dorsiflexors, leading to reduced balance or postural stability [17] and alterations in functional gait and mobility [15, 16]. Evidence from published literature suggests that structured physiotherapy or exercise interventions can help improve mobility and balance in individuals with DPN [18–20]. However, the goal of these studies has been to prevent falls in individuals with DPN, rather than improving HRQoL.

To our knowledge, only one previous study has examined the effect of exercise on HRQoL in individuals with DPN, reporting improvement in neuropathy-specific quality-of-life scores after 8 weeks of an aerobic exercise intervention [21]. However, the intervention was not designed to target functional performance; nor was this reported. In our previous work examining cross-sectional associations between DPN status, physical functioning and HRQoL, we found that functional measures, including functional performance, balance and balance confidence, partly mediated the observed association between DPN and lower HRQoL [22]. Other researchers have also reported that non-pain neuropathy symptoms such as unsteadiness and restrictions in daily activities are associated with increased fear of falling and psychological distress [23]. Targeted interventions to address these functional deficits, including balance confidence, may potentially improve both functional status and HRQoL. With this rationale, we conducted a randomised controlled trial to assess the effectiveness of an intervention focusing on lower-limb deficits that limit balance and functional mobility in individuals with DPN, with the primary aim of improving overall HRQoL and secondary aim of improving functional status.

## Methods

### Study design

The study was designed as a single-blind parallel-group randomised controlled trial comparing once-weekly home-based strength and balance training against standard medical therapy, with 2 months of intervention and an additional follow-up of 4 months. The National Healthcare Group Domain Specific Review Board and SingHealth Centralised Institutional Review Board reviewed and approved the study prior to study initiation. All participants provided written informed consent before any study procedure was started. The study was registered with the US National Library of Medicine Clinical Trials Registry (ClinicalTrials.gov registration no, NCT02115932).

### Participants

Recruitment for this study was conducted between July 2014 and October 2017 at outpatient clinics of five public sector institutions (four tertiary care hospitals and one primary care polyclinic). Individuals were eligible to participate in the study if they had physician-diagnosed type 2 diabetes, with peripheral neuropathy (defined by neurothesiometer reading greater than 25 V [24] and/or positive monofilament test in two or more sites in either foot [25] and/or Michigan Neuropathy Screening Instrument questionnaire score of 7 or greater [26]) and were between 40 and 79 years of age. Individuals were excluded from study participation if they met the following criteria: foot ulceration/infection/amputation; any contra-indication for physical activity or physiotherapy; non-diabetic neuropathy (e.g. as indicated by vitamin B_12_ deficiency, hypothyroidism [as measured during screening], and alcohol abuse [assessed by AUDIT questionnaire [27] and defined as ingestion of 100 ml or more of ethyl alcohol per day for 3 years or more]); non-diabetes and non-neuropathy-related orthopaedic, surgical or medical conditions affecting functional mobility and balance or if individuals were non-ambulatory for any reason.

All eligible and consenting participants underwent baseline assessment of HRQoL and functional status before being randomised to control or intervention arms by an independent statistician, who was not involved in the conduct or analysis of the trial. Random permuted blocks with randomly varying block size of 4 and 6 were generated assuming a 1:1 treatment allocation stratified by recruitment site. For each participant, the assignment was directly conveyed by the statistician to the intervention physiotherapist.

### Intervention

Individuals assigned to the intervention arm received once-weekly balance retraining and strengthening interventions guided by a physiotherapist for 8 weeks. Training sessions were conducted at the participant’s home or other place designated by the participant. Each session was a one-on-one session with the individual participant and the physiotherapist. The training intervention was developed by the research team based on prior research evidence that individuals with DPN have the following conditions: (1) diminished muscle strength at the ankle and below [15, 28]; (2) reduced ankle mobility and range of motion [15, 16] and (3) postural instability or reduced balance during standing and while walking [17, 29]. The intervention included the following components:

- Range of motion exercises: passive movements to the extent possible of the knee (flexion–extension), ankle (dorsi–plantar flexion), forefoot (inversion–eversion) and toe (flexion–extension, adduction–abduction) joints
- Muscle strengthening exercises: active movements against resistance (using a theraband) at the knee (flexion–extension), ankle (dorsi–plantar flexion), forefoot (inversion–eversion) and toe (flexion–extension, adduction–abduction) joints
- Exercises for improving static balance: single leg stance, tandem leg stance, toe and heel stance
- Exercises for improving dynamic balance: tandem walk, sideways walk, backward walk
- Endurance exercises: e.g. sitting-to-standing minihops, brisk walking

All sessions began with a 5 min pre-exercise warm-up of gentle stretches and ended with a 5 min cool-down of slow walking. The intensity of the exercises was gradually increased based on participant performance during the course of the intervention. In any session, participant report of pain, cramps, fatigue or any discomfort during the session were indications to stop the session, though this did not happen in any session for any participant. In addition, participants were encouraged to perform the exercises taught at least three times a week and up to once per day. The frequency of self-exercise sessions was recorded by the physiotherapist on the weekly visit to the participant. During the 8 weeks of intervention, participants reported performing intervention exercises on a median of 25 days (of a possible total of 56 days), with a range of 0–49 days.

Participants in the control arm received routine clinical care from their healthcare providers as per recommended clinical care guidelines. These participants received no specific care/intervention from the study team but were followed up for assessments at the same time intervals as those in the intervention arm.

### Assessments

All participants underwent assessment through planned clinic visits at three time points (at baseline and at 2 months and 6 months after baseline). At each time point, HRQoL was assessed using the SF-36v2 and EQ-5D-5L. The SF-36v2 is a 36-item generic HRQoL instrument that has been validated for use in Singapore [30]. All SF-36v2 scores were weighted using Singapore general population data before computing individual domain scores, the physical component summary (PCS) score and the mental component summary (MCS) score. The EQ-5D-5L is a generic HRQoL instrument with five items, from which a summary index score was calculated using the Japanese value set [31].

Functional assessment included measurement of functional mobility, static balance, muscle strength and range of motion. Functional mobility was assessed through the timed up-and-go (TUG) test, five times sit-to-stand (FTSTS) test, functional reach and balance confidence. The TUG test measures the time taken by a participant to stand from a sitting position, walk 3 m, return and sit back down and is a measure of mobility. The FTSTS measures the time taken by a participant to switch from sitting-to-standing five times in a row and is a test of functional strength. Functional reach measures the distance a participant can reach forward with his or her arm outstretched while standing and is a test of balance. For each test, participants completed a practice run before the actual measurement. The activities-specific balance confidence (ABC) scale was used to measure balance confidence. The ABC scale is a 16-item self-administered questionnaire, with each item assessing participants’ confidence (from 0% to 100%) in undertaking a specific task without losing balance. Individual item scores were averaged to compute the total ABC score.

Average body sway velocity, as a measure of static balance, was measured using a balance platform (Accugait; AMTI, Watertown, MA, USA). Participants were instructed to stand on the balance platform with eyes closed for 2 min. The mean from two rounds of testing was used.

Muscle strength at the ankle during dorsiflexion was measured with the participant seated and the knee extended, using a hand-held dynamometer (micro FET3; Hoggan Scientific, Salt Lake City, UT, USA) placed on the dorsum of the foot. Range of motion for dorsiflexion–plantar flexion at the ankle was measured using a hand-held inclinometer (MicroFET3; Hoggan Scientific) positioned on the dorsum of the foot, with the participant seated, the knee extended and the ankle fully plantar-flexed at the start. Range of motion for flexion–extension at the knee was measured using the hand-held inclinometer placed on the lower third of the back of the leg of interest, with the participant standing and starting with a fully extended knee. For each test, the mean from two rounds of testing was used after an initial trial. Muscle strength and range of motion were assessed on both lower limbs.

All assessments were conducted by a trained research assistant who was blinded to the participants’ randomisation status.

### Primary and secondary outcomes

The primary outcome was change in HRQoL between treatment arms over the 6 months as measured by the PCS and EQ-5D-5L index scores. Secondary outcomes were change in other domains of the SF-36v2, functional mobility, static balance, muscle strength and range of motion between the arms over the 6 months.

### Statistical analysis

We postulated that, on average, there would be a 4-point difference in PCS score between the intervention vs control arms, with an SD of 13. This was based on our previous work where this magnitude of difference was observed between individuals with DPN and individuals with diabetes only [2]. We hypothesised that the intervention would be able to increase PCS score by a similar magnitude if all of the observed difference was due to poor functional status. Assuming a repeated measures study design with one baseline and two post-intervention measurements and a within-subject correlation of 0.6, a total of 200 individuals (i.e. 100 per arm) would be required, based on a two-sided α of 0.05 and a 90% power. Further accounting for 10% loss to follow-up, a total sample size of 220 would be anticipated.

Means and SD (or range in the case of age) were used to describe participant characteristics that were continuous, whereas frequencies and percentages were used to summarise categorical variables. Measurements of muscle strength and range of motion were highly correlated between the two sides of participants. Hence, all measurements used in this analysis were from the right lower limb for the purpose of standardisation. Random intercept linear mixed models with repeated measures at 2 months and 6 months were used to compare the outcomes between the intervention and control groups, adjusting for the respective baseline covariate. We additionally adjusted for the potential confounding effects of sex and time.

Furthermore, we examined whether the changes in HRQoL and functional variables observed were clinically important. A threshold of 0.2 × SD at baseline (SD_b_) was used to define the minimum clinically important difference for each outcome of interest [32]. Therefore, any mean difference between intervention and control groups in change in outcome variable(s) ≥0.2 × SD_b_ was deemed to be clinically important.

All analysis was conducted based on the principle of intention-to-treat, using Stata (version 15; StataCorp, College Station, TX, USA), assuming a two-sided test at the 5% level of significance.

## Results

**Fig. 1:**
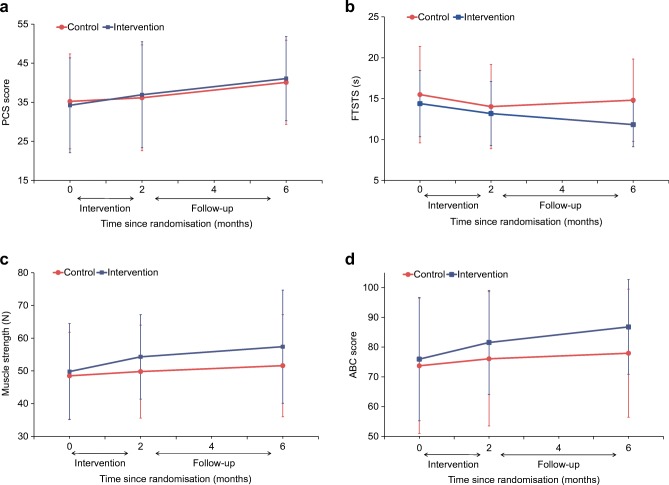
Time trends in selected outcomes in intervention and control groups: (**a**) PCS score (primary outcome); (**b**) FTSTS (secondary outcome); (**c**) muscle strength at right ankle (secondary outcome); and (**d**) ABC score (secondary outcome)

A total of 143 participants were enrolled in the study, with 70 randomised to the intervention arm and 73 to the control arm. The CONSORT diagram of participant recruitment and flow is presented in electronic supplementary material (ESM) Fig. 1. Three intervention and six control arm participants were lost to follow-up at 2 months, while two intervention and three control arm participants were lost to follow-up at 6 months. This left 67 participants in each arm available for the intention-to-treat analysis.

The mean age of enrolled participants was 62 years, 80 (56%) were women and most (110, 77%) were of South Asian ethnicity. Mean duration of diabetes at enrolment was 15.3 (SD 10.7) years, with hypertension, hypercholesterolaemia, heart disease and retinopathy being the most common comorbidities reported. Most participants were not symptomatic for DPN; only four participants (two in each arm) had Michigan Neuropathy Screening Instrument history scores of 7 or more. The most common symptoms were leg cramps and numbness. At enrolment, mean BMI was 28.4 (SD 5.7) kg/m^2^ and mean HbA_1c_ was 69 mmol/mol (8.5%). Participants in the intervention and control arms were comparable in terms of demographic and clinical characteristics, except for there being a higher proportion of women in the control arm (ESM Table 1). Table 1 shows the distribution of key outcomes of interest at baseline across both arms, where again both groups were similar.

**Table 1:** HRQoL and functional scores of study participants at baseline

Characteristic	Intervention (*n* = 70)	Control (*n* = 73)	All participants (*n* = 143)
PCS score	34.1 (12.2)	35.1 (11.9)	34.6 (12.0)
EQ-5D-5L index score	0.73 (0.16)	0.71 (0.17)	0.72 (0.16)
Functional status			
TUG test, s	10.9 (3.9)	12.2 (4.8)	11.6 (4.4)
FTSTS test, s	14.4 (4.0)	15.6 (5.8)	15.0 (5.0)
Functional reach, cm	24.3 (7.0)	23.7 (6.9)	24.0 (6.9)
Total ABC score, %	76.3 (20.5)	73.3 (22.6)	74.8 (21.6)
Body sway velocity, eyes closed, mm/s	1.6 (1.3)	1.6 (1.1)	1.6 (1.2)
Muscle strength, right ankle, N	49.8 (14.7)	48.5 (13.3)	49.4 (13.8)
Range of motion,			
Right ankle	78.0 (9.6)	78.5 (10.8)	78.2 (10.2)
Right knee	104.9 (15.9)	101.7 (19.8)	103.3 (18.0)

All values are mean (SD)

Measurements of muscle strength and range of motion were highly correlated between the right and the left sides, therefore only right-side measurements are reported

### Primary outcomes

When comparing change in primary outcomes over the 6 months, we found no significant difference in either PCS score (mean difference 1.56 [95% CI −1.75, 4.87]; *p* = 0.355) or EQ-5D-5L index score (mean difference 0.02 [95% CI −0.01, 0.06]; *p* = 0.175) between the groups (Table 2). We examined changes in other domains of the SF-36v2 as secondary outcomes. There was significant improvement in the body pain domain in the intervention group compared with the control group (mean difference 5.14 [95% CI 2.05, 8.23]; *p* = 0.001). Improvement was also noted in the general health domain (mean difference 2.36 [95% CI −0.28, 4.99]; *p* = 0.080), though this did not reach statistical significance.

**Table 2:** Mean differences (95% CI) in HRQoL (primary outcomes) between intervention and control groups

Characteristic	Mean difference	95% CI	*p* value
PCS score			
Model 1	1.56	−1.75, 4.87	0.355
Model 2	0.92	−2.38, 4.20	0.586
EQ-5D-5L index score			
Model 1	0.02	−0.01, 0.06	0.175
Model 2	0.02	−0.02, 0.05	0.290
Physical functioning			
Model 1	−0.19	−3.83, 3.46	0.920
Model 2	−1.15	−4.65, 2.36	0.521
Role physical			
Model 1	−0.18	−3.82, 3.46	0.923
Model 2	−0.82	−4.39, 2.75	0.654
Body pain			
Model 1	5.14	2.05, 8.23	0.001
Model 2	4.94	1.76, 8.11	0.002
General health			
Model 1	2.36	−0.28, 4.99	0.080
Model 2	2.23	−0.39, 4.85	0.095
Vitality			
Model 1	0.71	−2.11, 3.52	0.623
Model 2	0.39	−2.35, 3.13	0.780
Social functioning			
Model 1	1.44	−1.26, 4.14	0.295
Model 2	1.28	−1.35, 3.91	0.338
Role emotional			
Model 1	1.45	−2.32, 5.22	0.450
Model 2	1.03	−2.69, 4.76	0.587
Mental health			
Model 1	1.70	−1.16, 4.56	0.243
Model 2	1.06	−1.64, 3.75	0.442
MCS score			
Model 1	1.36	−0.89, 3.60	0.236
Model 2	1.19	−1.02, 3.41	0.290

Model 1 was a random intercept mixed model with intervention and one pre-measurement; model 2 was a random intercept mixed model, adjusted for time, baseline covariate and sex

### Secondary outcomes

We also compared change in the secondary outcomes of functional mobility, static balance, muscle strength and range of motion over the 6 months between intervention and control groups (Table 3). There were significant improvements in performance in the TUG (mean difference −1.14 [95% CI −2.18, −0.10] s; *p* = 0.032) and FTSTS tests (mean difference −1.31 [95% CI −2.12, −0.51] s; *p* = 0.001), ABC score (mean difference 6.17 [95% CI 1.89, 10.44]; *p* = 0.005), muscle strength at ankle (mean difference 4.18 [95% CI 0.4, 7.92] N; *p* = 0.031) and range of motion at knee (mean difference 6.82 [95% CI 2.87, 10.78]°; *p* = 0.001). There was also significant loss of range of ankle motion in the intervention group compared with the control group (mean difference −3.17 [95% CI −5.75, −0.58]°; *p* = 0.016), which corresponded to an increase in ankle muscle strength in the intervention group. These results were not materially altered after adjusting for sex and the effect of time, except for ankle muscle strength and TUG test result. The improvements observed in TUG test, FTSTS test, ABC, ankle muscle strength and range of knee motion were also greater than the calculated minimum clinically important difference for each measure.

**Table 3:** Mean differences (95% CI) in functional measures (secondary outcomes) between intervention and control groups

Characteristic	Mean difference	95% CI	*p* value
TUG, s			
Model 1	−1.14	−2.18, −0.10	0.032
Model 2	−1.02	−2.07, 0.02	0.054
FTSTS, s			
Model 1	−1.31	−2.12, −0.51	0.001
Model 2	−1.21	−2.01, −0.42	0.003
Functional reach, cm			
Model 1	0.50	−1.29, 2.28	0.585
Model 2	0.19	−1.59, 1.97	0.836
Total ABC score, %			
Model 1	6.17	1.89, 10.44	0.005
Model 2	5.50	1.31, 9.68	0.010
Body sway velocity, eyes closed, mm/s			
Model 1	0.17	−0.02, 0.36	0.087
Model 2	0.19	−0.01, 0.39	0.065
Muscle strength, right ankle, N			
Model 1	4.18	0.4, 7.92	0.031
Model 2	3.69	0.00003, 7.43	0.050
Range of motion, right ankle,			
Model 1	−3.17	−5.75, −0.58	0.016
Model 2	−3.20	−5.83, −0.57	0.017
Range of motion, right knee,			
Model 1	6.82	2.87, 10.78	0.001
Model 2	6.48	2.38, 10.59	0.002

Model 1 was a random intercept mixed model with intervention and one pre-measurement; model 2 was a random intercept mixed model, adjusted for time, baseline covariate and sex

### Time trends in primary and secondary outcomes

We plotted the raw values of selected outcomes over time for both groups to understand changes in these variables during the intervention and post-intervention phases. PCS scores improved by around five points over the entire study duration in both groups, from 34.2 ± 11.9 at baseline to 36.9 ± 12.8 at 2 months and 41.1 ± 11.6 at 6 months in the intervention group, and 35.2 ± 12.1 at baseline to 36.2 ± 13.6 at 2 months and 40.1 ± 10.8 and 6 months in the control group (Fig. 1a). Performance in the FTSTS test improved in the intervention group from 14.4 ± 4.1 s at baseline to 13.2 ± 3.9 s at 2 months and 11.8 ± 2.7 s at 6 months (Fig. 1b). In contrast, FTSTS results in the control group remained stable, with times of 15.5 ± 5.9 s at baseline, 14.0 ± 5.2 s at 2 months and 14.8 ± 5.0 s at 6 months. At baseline, ankle muscle strength was 49.8 ± 14.7 N in the intervention group and 48.5 ± 13.3 N in the control group (Fig. 1c). Strength increased at 2 months to 54.3 ± 12.9 N and 49.8 ± 14.2 N in the intervention and control groups, respectively. At 6 months, muscle strength at ankle was 57.4 ± 17.3 N in the intervention group and 51.6 ± 15.6 N in the control group. Participants in the intervention group showed improvement in ABC score from 76.0 ± 20.7 at baseline to 81.6 ± 17.5 at 2 months and 86.8 ± 15.9 at 6 months (Fig. 1d). Corresponding values for baseline, 2 and 6 months were 73.7 ± 22.7, 76.1 ± 22.6 and 77.9 ± 21.5 for participants in the control group.

### Additional analyses

To examine whether improvements in functional status had any effect on HRQoL, we also conducted subgroup analysis on participants in the intervention arm using generalised estimating equations (ESM Table 2). Improvements in TUG test, FTSTS test and ABC scores and range of motion at knee were associated with greater improvements in EQ-5D-5L index scores.

No adverse events due to study participation or study intervention were reported. None of the participants on either arm developed foot ulceration or infection during the study period. Four participants (one intervention, three control) reported falling in the past 4 weeks at baseline, while three (two intervention, one control) and five (two intervention, three control) reported falling at 2 and 6 months, respectively. None of the falls occurred during the intervention exercises and no injuries were reported as a result of the falls.

## Discussion

The current study found no significant difference in overall HRQoL scores between intervention and control arms after 2 months of structured strength and balance training in individuals with DPN. However, we found significantly greater improvement in the body pain domain of HRQoL in the intervention arm as compared with the control arm. In addition, there were significant improvements in several functional status variables in the intervention arm compared with the control arm. These included statistically significant and clinically meaningful improvements in functional task performance, balance confidence, range of motion at knee and muscle strength at ankle. These improvements were sustained for up to 4 months after the end of the intervention.

Our previous cross-sectional study demonstrated significant differences in both EQ-5D-5L scores and functional status between diabetic individuals with and without DPN. Functional measures, specifically FTSTS test results and balance confidence, partially mediated the association between DPN status and HRQoL [22]. In our current study, we did not demonstrate any effect of our intervention on overall HRQoL scores, either PCS (SF-36v2) or EQ-5D-5L, despite significant gains in FTSTS performance and balance confidence. Based on the previous analysis, one-unit change in balance confidence and FTSTS performance would result in a 0.005 increase in EQ-5D-5L. The magnitude of change observed in EQ-5D-5L scores in our current trial is consistent with this, with greater improvement in EQ-5D-5L scores with improvement in functional measures in participants in the intervention arm. These results suggest that substantially larger changes in functional measures would be needed to meaningfully change HRQoL in these individuals. More intensive physical therapy over a longer period of time may be needed to effect improvement in HRQoL. However, more intensive physical interventions may potentially lead to adverse events, including pain, muscle strain and even ulceration [33, 34]; careful assessment of the risks vs benefits would be needed for any individual before prescription of intensive physical therapy.

Dixit et al have previously reported significant improvements in overall quality-of-life scores, as well as in pain, sensory motor symptoms, activities of daily living and social relationships scores with 8 weeks of aerobic exercise [21]. This is in contrast to our findings. However, Dixit et al used a neuropathy-specific instrument to assess quality of life, which would be more sensitive to change compared with the generic instruments we have used. Nevertheless, disease-specific instruments do not allow for a comparison between diseases, which is important for prioritising interventions for resource allocation; this consideration governed our choice of instrument.

HRQoL scores in our study improved over time in both groups and to a similar extent. This may possibly be due to the Hawthorne effect [35] as participants on both arms had similar contact and support from the study team during the duration of the study. Other treatments and external factors beyond the study may also affect how an individual’s HRQoL changes over time. Among the specific domains of HRQoL, body pain was the only domain that showed significant improvement with intervention. It is possible that the improved physical conditioning in these individuals may have reduced the effect of pain on daily life and activities [36, 37]. However, it needs to be noted that the body pain domain is not specific to neuropathic pain and participants may have had pain due to other comorbidities that responded to the intervention.

The change in functional variables with the intervention is in line with previously published literature. A variety of exercise interventions enhanced postural stability, functional performance and lower-limb strength in a meta-analysis of such interventions in individuals with diabetes [38]. Similar improvements have been reported in individuals with DPN [33]. Richardson et al showed significant improvements in unipedal stance time, functional reach and tandem stance time in ten individuals with peripheral neuropathy who underwent a 3 week exercise intervention as compared with control individuals who did not receive the intervention [18]. The exercises included range of motion, inversion–eversion and toe and heel raises performed daily. However, there was no significant difference in balance confidence between intervention and control groups [18]. Allet et al demonstrated significant improvements in walking speed and gait variability in challenging terrains, as well as improvements in balance, strength and mobility in 35 diabetic individuals who received physiotherapy training over 12 weeks compared with 36 control individuals who did not receive physiotherapy [19, 20]. The physiotherapy intervention consisted of twice weekly group sessions of 60 min, consisting mainly of gait and balance exercises. Improvements in the intervention group were significant both at the end of the 12 week period and at follow-up at 6 months [19, 20]. However, small sample size has been a consistent limitation of these studies, and our larger-scale trial provides supportive evidence for the positive effect of physical therapy on functional status in DPN.

One important functional measure that improved as a result of the intervention was balance confidence. Low balance confidence has been associated with greater physical difficulties and lower HRQoL [22, 39]. More importantly, low balance confidence has been shown to predict poorer mobility in the future [40]. Balance confidence may determine the nature, duration and intensity of physical activities an individual undertakes on a daily basis and a decline in confidence, either due to a previous fall or the fear of falling, may place an individual on a downward spiral of declining physical functioning and further deteriorating balance confidence. Ours is the first randomised controlled trial to demonstrate the effectiveness of structured physical therapy in improving balance confidence in individuals with DPN.

Some limitations of the study need to be acknowledged. As DPN was defined using only simple clinical assessments, the findings may not be applicable to those with early or small-fibre neuropathy. We chose these assessments as they are used routinely in clinics to identify individuals at risk of foot problems due to neuropathy. In addition, we did not assess the severity of DPN in these individuals as the study primarily focused on functional improvement in DPN rather than on reduction of neuropathy progression. Hence, we were unable to comment on the potential effect of the intervention on DPN severity. We used two commonly used generic HRQoL instruments, which may not capture the impact of DPN with sufficient granularity. Therefore, we may have missed subtle changes in HRQoL that a disease-specific instrument may have identified. Generic instruments were chosen in this study as we have demonstrated significant reductions in HRQoL in DPN using both SF-36v2 and EQ-5D-5L previously. In addition, use of generic instruments allows the comparison of intervention effect in HRQoL between conditions. The sample size achieved was smaller than the target sample size, raising the concern that no significant differences in primary outcomes were found due to inadequate sample size. However, the magnitude of change in HRQoL outcomes observed was much smaller than anticipated during sample size calculations and was too small to have been significant even if the target sample size had been achieved. The magnitude of change in HRQoL observed in our trial is consistent with the strength of association between HRQoL and functional status observed previously. Hence, it appears more likely that the failure to detect a difference in the primary outcome in our study is due to lack of effect on quality of life by an intervention of this intensity, rather than a lack of power. The challenge to achieving target sample size was mainly the relatively small pool of patients with clinically evident neuropathy without active foot problems, foot deformities or prior amputations. The bulk of the patients with clinically evident neuropathy had either existing foot conditions or had severe comorbid conditions that precluded them from participation.

The use of a randomised controlled trial design, blinding of the assessor and the low proportion of participants lost to follow-up are key strengths of the study. The study intervention consisted of easy to understand exercises, was delivered at participants’ homes, was individualised to progress and resulted in significant improvements in a number of functional variables, which is another major strength. Other strengths are the comprehensive assessment of functional status and HRQoL, as well as the follow-up of participants for an additional 4 months post-intervention, allowing the examination of sustained improvements in outcomes of interest.

In conclusion, we have demonstrated that short-term structured strength and balance training resulted in sustained improvements in functional status at 6 months in individuals with DPN but that the magnitude of improvement in functional status did not appear to be sufficiently large to impact overall HRQoL. Longer-term and more intensive interventions may be needed to influence HRQoL in these individuals. Nonetheless, an intervention of this nature may help to preserve functional status, improve balance confidence and reduce the likelihood of falls and injuries in individuals with DPN. In addition to improving glycaemic control, the only specific treatments for DPN available in clinical practice are for pain relief [41]. However, only a subset of individuals with DPN have painful neuropathy, while loss of functional capacity affects almost all individuals. Therefore, such an intervention can be a useful treatment option for patients with DPN in clinical practice, especially for those without pain symptoms, and this should be evaluated in future studies.

## Electronic supplementary material

ESM (PDF 113 kb)

## Data Availability

The datasets generated during and/or analysed during the current study are available from the corresponding author on reasonable request.

## Abbreviations

ABC: Activities-specific balance confidence
DPN: Diabetic peripheral neuropathy
FTSTS: Five times sit-to-stand test
HRQoL: Health-related quality of life
MCS: Mental component summary
PCS: Physical component summary
TUG: Timed up-and-go
