# Supplementary material for: Short-term strength and balance training does not improve quality of life but improves functional status in individuals with diabetic peripheral neuropathy: a randomised controlled trial
Source: Diabetologia. 2019 Aug 29;62(12):2200–10. doi: 10.1007/s00125-019-04979-7 (PMC6861346; doi:10.1007/s00125-019-04979-7)

ESM Table 1. Demographic and clinical characteristics of study participants at baseline

Characteristic	Intervention (n = 70)	Control (n = 73)	All patients (n = 143)
**Age (years)**			
Mean	61.9	62.3	62.1
Range	47.6 – 78.4	43.2 – 78.4	43.2 – 78.4
**Gender (%)**			
Male	36 (51.4)	27 (37.0)	63 (44.1)
Female	34 (48.6)	46 (63.0)	80 (55.9)
**Ethnicity (%)**			
South Asian	56 (80.0)	54 (74.0)	110 (76.9)
Other	14 (20.0)	19 (26.0)	33 (23.1)
**Currently married (%)**	50 (71.4)	44 (60.3)	94 (65.7)
**At least secondary education (%)**	33 (47.1)	37 (50.7)	70 (49.0)
**Employed (%)**	34 (48.6)	32 (43.8)	66 (46.2)
**Monthly household income ≥ $2,000 (%) ***	15 (26.3)	14 (26.4)	29 (26.4)
**Housing type (%)**			
1–2 room	33 (54.1)	32 (50.8)	65 (52.4)
3-4 room	28 (45.9)	29 (46.0)	57 (46.0)
5-room or above	0 (0.0)	1 (1.6)	1 (0.8)
**Diabetes duration (years)**	13.8 (10.3)	16.8 (11.1)	15.3 (10.7)
**Other comorbidities**			
**Hypertension**	46 (65.7)	54 (74.0)	100 (69.9)
**Hypercholesterolemia**	45 (64.3)	52 (71.2)	97 (67.8)
**Heart disease**	19 (27.1)	14 (19.2)	33 (23.1)
**Kidney disease**	1 (1.4)	2 (2.7)	3 (2.1)
**Stroke**	2 (2.9)	5 (6.8)	7 (4.9)
**Peripheral arterial disease**	4 (5.7)	6 (8.2)	10 (7.0)
**Retinopathy**	20 (28.6)	26 (35.6)	46 (32.2)
**Arthritis**	8 (11.4)	8 (11.0)	16 (11.2)
**Michigan Neuropathy Screening Instrument history score**			
**>=7**	2 (2.9)	2 (2.7)	4 (2.8)
**>=4**	23 (32.9)	31 (42.5)	54 (37.8)
**On DPN medication, n (%)**	2 (2.9)	3 (4.1)	5 (3.5)
**Falls in the past 4 weeks , n (%)**	1 (1.4)	3 (4.1)	4 (2.8)
**Mean BMI, kg/m^2 (SD)**	28.4 (5.6)	28.4 (5.9)	28.4 (5.7)
**Baseline HbA1c (mmol/mol) [%]**	67 (19.7) [8.3 (1.8)]	70 (19.7) [8.6 (1.8)]	69 (19.7) [8.5 (1.8)]
**HbA1c at 2-month (mmol/mol) [%]**	64 (17.5) 8.0 (1.6)	68 (18.6) [8.4 (1.7)]	66 (18.6) [8.2 (1.7)]
**HbA1c at 6-month (mmol/mol) [%]**	66 (17.5)[8.2 (1.6)]	70 (23.0) [8.6 (2.1)]	68 (19.7) [8.4 (1.8)]

* Thirty-three observations with missing monthly household income.

ESM Table 2. Association between HRQoL and functional status among intervention group

Characteristic	Effect estimate	95% CI	p-value
**Timed up and go (seconds)**			
Model 1	-0.017	(-0.025, -0.009)	<0.001
Model 2	-0.015	(-0.023, -0.006)	0.001
**Five times Sit-to-stand (seconds)**			
Model 1	-0.011	(-0.019, -0.003)	0.010
Model 2	-0.006	(-0.015, 0.003)	0.170
**Functional reach (cm)**			
Model 1	-0.003	(-0.007, 0.002)	0.260
Model 2	-0.002	(-0.006, 0.003)	0.397
**Total ABC score (%)**			
Model 1	0.004	(0.003, 0.006)	<0.001
Model 2	0.003	(0.002, 0.005)	<0.001
**Body sway velocity, eyes closed (mm/s)**			
Model 1	-0.027	(-0.066, 0.012)	0.174
Model 2	-0.007	(-0.045, 0.031)	0.720
**Muscle strength, right ankle (N)**			
Model 1	0.001	(-0.0004, 0.003)	0.140
Model 2	0.0002	(-0.002, 0.002)	0.921
**Range of motion, right ankle (degrees)**			
Model 1	-0.002	(-0.004, 0.001)	0.208
Model 2	-0.001	(-0.004, 0.001)	0.360
**Range of motion, right knee (degrees)**			
Model 1	0.002	(0.001, 0.004)	0.014
Model 2	0.001	(0, 0.003)	0.143

ABC: activities-based balance confidence
Dependent variable: EQ-5D-5L index score
Note: Model 1 – GEE with gaussian distribution and unstructured correlation, adjusted with baseline measurement of functional status.
Model 2 – GEE with gaussian distribution and unstructured correlation, adjusted for time, baseline measurement of functional status and gender.

ESM Fig. 1. CONSORT diagram of participant flow

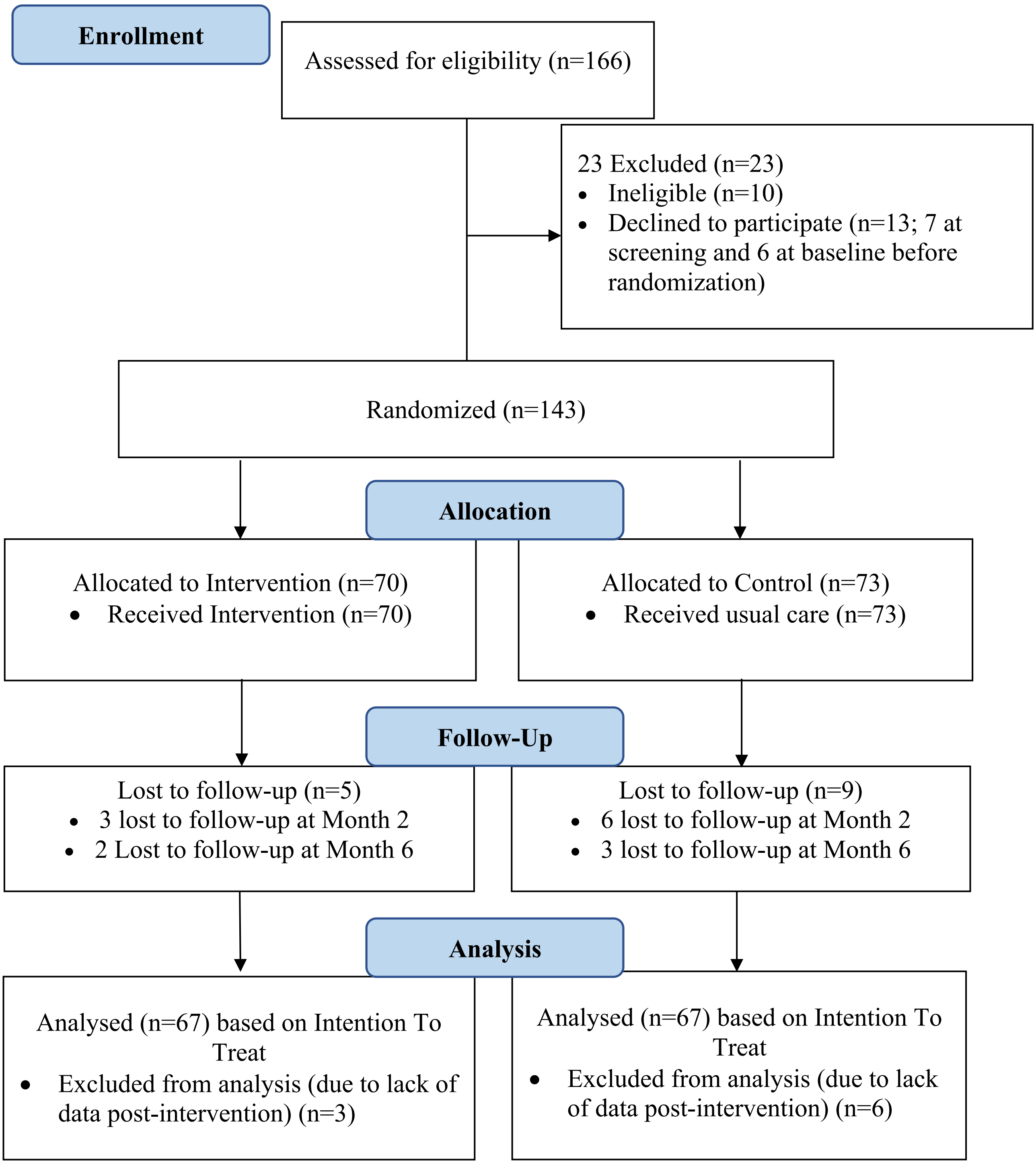

Supplement: Supplementary file 1 — (PDF 113 kb) [file 125_2019_4979_MOESM1_ESM.pdf]
